# Green Chemometric Determination of Cefotaxime Sodium in the Presence of Its Degradation Impurities Using Different Multivariate Data Processing Tools; GAPI and AGREE Greenness Evaluation

**DOI:** 10.3390/molecules28052187

**Published:** 2023-02-26

**Authors:** Yasmine Ahmed Sharaf, Adel Ehab Ibrahim, Sami El Deeb, Rania Adel Sayed

**Affiliations:** 1 Pharmaceutical Analytical Chemistry Department, Faculty of Pharmacy, Zagazig University, Zagazig 44511, Egypt; 2 Natural and Medical Sciences Research Center, University of Nizwa, P.O. Box 33, Birkat Al Mauz, Nizwa 616, Oman; 3 Pharmaceutical analytical chemistry Department, Faculty of Pharmacy, Port-Said University, Port-Said 42526, Egypt; 4 Institute of Medicinal and Pharmaceutical Chemistry, Technische Universitaet Braunschweig, 38106 Braunschweig, Germany

**Keywords:** cefotaxime, chemometry, multilevel experimental design, stability-indicating, greenness assessment

## Abstract

Four eco-friendly, cost-effective, and fast stability-indicating UV-VIS spectrophotometric methods were validated for cefotaxime sodium (CFX) determination either in the presence of its acidic or alkaline degradation products. The applied methods used multivariate chemometry, namely, classical least square (CLS), principal component regression (PCR), partial least square (PLS), and genetic algorithm-partial least square (GA-PLS), to resolve the analytes’ spectral overlap. The spectral zone for the studied mixtures was within the range from 220 to 320 nm at a 1 nm interval. The selected region showed severe overlap in the UV spectra of cefotaxime sodium and its acidic or alkaline degradation products. Seventeen mixtures were used for the models’ construction, and eight were used as an external validation set. For the PLS and GA-PLS models, a number of latent factors were determined as a pre-step before the modelsʹ construction and found to be three for the (CFX/acidic degradants) mixture and two for the (CFX/alkaline degradants) mixture. For GA-PLS, spectral points were minimized to around 45% of the PLS models. The root mean square errors of prediction were found to be (0.19, 0.29, 0.47, and 0.20) for the (CFX/acidic degradants) mixture and (0.21, 0.21, 0.21, and 0.22) for the (CFX/alkaline degradants) mixture for CLS, PCR, PLS, and GA-PLS, respectively, indicating the excellent accuracy and precision of the developed models. The linear concentration range was studied within 12–20 μg mL^–1^ for CFX in both mixtures. The validity of the developed models was also judged using other different calculated tools such as root mean square error of cross validation, percentage recoveries, standard deviations, and correlation coefficients, which indicated excellent results. The developed methods were also applied to the determination of cefotaxime sodium in marketed vials, with satisfactory results. The results were statistically compared to the reported method, revealing no significant differences. Furthermore, the greenness profiles of the proposed methods were assessed using the GAPI and AGREE metrics.

## 1. Introduction

Drug stability is a critical issue during the screening of pharmaceutical dosage forms. Stability testing and impurity profiling are important steps in the quality control of drug products to ensure accurate and appropriate delivery of drug dose therapy to patients. The source of drug impurities may originate during the synthesis procedure or as degradation products during the transportation and shelf-life of pharmaceutical products [1]. Such chemical impurities may cause changes in the pharmacological and toxicological properties of the active drug form and subsequently affect its safety and efficacy [2].

**Figure 1:**
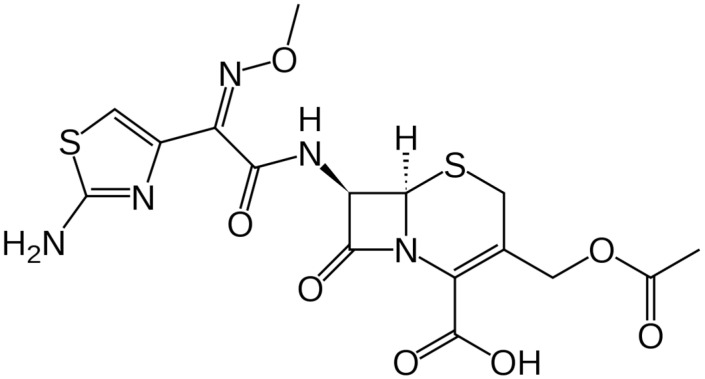
CFX chemical structure.

Meanwhile, the parenteral antibiotic dosage form accounts for about 50% of the total worldwide antibiotic sales [3]. The total defined daily antibiotic dose consumption has increased substantially during the past few years [4,5], among which third-generation cephalosporins represent about 20% of antibiotic consumption [3]. Cephalosporins are a key antimicrobial class for the treatment of infectious diseases in both humans and animal species [6], of which the third generation could account for about 50% of total cephalosporin sales [3]. These figures can give an abrupt estimation of the huge size of the production of cephalosporins’ pharmaceutical products. Cefotaxime (CFX) is a semi-synthetic antibiotic belonging to the third generation cephalosporin class (the chemical structure is shown in Figure 1). It is a broad-spectrum antimicrobial agent [7] that has been listed and classified as a critically important antimicrobial agent [8,9]. 

The literature review showed some published methods that were applied for the simultaneous determination of CFX in a mixture of similarly structured cephalosporins using either a spectrophotometric method [10,11] or Raman spectroscopy [12]. Being parentally administered, CFX must show good stability in solutions with a pH between 4.5 and 6.5 with no degradation products observed [13]. On the other hand, at pH values of 2 and 10, higher degradation rates are observed due to hydrogen or hydroxide ion catalysis [14]. Therefore, it is of great importance to establish methods for the assay of CFX in the presence of either its acidic or alkaline degradation products. Several chromatographic methods have been reported for the quantitative determination of CFX in the presence of its degradation products, including HPTLC [2,15,16] and HPLC [14,17,18]. The British Pharmacopoeia [19] describes a time-consuming gradient HPLC method for the impurity profiling of CFX that involves more than 60 min of run time. Only a few spectrophotometric methods were reported for CFX determination in the presence of its degradation impurities [14,20,21].

Several analytical techniques are currently being used to determine the stability of different chemical drug molecules. UV-VIS spectrometry is one of the most widely used techniques for stability-indicating methods, as it can achieve the best compromise between ecological safety and maintaining the method’s efficiency and quality, especially when compared to the other chromatographic techniques. This advantage could be attributed to the low solvent consumption and a reduction in the amount of waste generated [22]. Additionally, the spectrophotometric technique does not require expensive instrumentation, which makes it an economic and cost-effective technique. However, the strongly overlapping analytesʹ spectra are the main problem facing research analysts while using a UV-VIS spectroscopic technique in multicomponent analysis [23]. Therefore, the multivariate methods were an excellent solution to this problem, as they represented a mathematical resolution of severely overlapped spectra, unlike the conventional univariate spectrophotometric techniques, which suffer from a lack of resolution [24,25]. Accordingly, multivariate chemometric methods were applied for the simultaneous determination of several multicomponent mixtures [26,27,28]. Moreover, the stability-indicating multivariate chemometric methods could be applied for the determination of several analytes in the presence of their degradation products [29,30,31]. In CLS, a multiple linear regression is applied to Beer’s law to construct the K-matrix. CLS is a direct method requiring full identification of the components in the training matrix. However, PLS is an indirect model that requires only knowing the concentration of the substance of interest [32]. On the other hand, PCR and PLS have excellent capabilities to analyze the highly overlapping spectra. Moreover, they can be highly tolerant to noise and interfering issues. Hence, PCR and PLS are the most frequently utilized multivariate chemometric methods [33]. A GA-PLS approach is also used for wavelength selection to properly minimize the number of selected wavelengths, leading to fitness maximization and error reduction [34].

Hence, the main aim of the present work was to develop four simple green chemometric methods that are stability-indicating for the determination of CFX in the presence of its acidic or alkaline degradation products. The developed methods are green, simple, cost-effective, sensitive, and accurate, and they can be used for routine analysis of CFX in pharmaceutical research and quality control laboratories.

## 2. Materials and Methods

### 2.1. Materials and Reagents 

Pure cefotaxime sodium was kindly supplied by the Egyptian International Pharmaceutical Industries Co. (EIPICo., Tenth of Ramadan city, Egypt). Hydrochloric acid (HCl) and sodium hydroxide (NaOH) were of analytical grades and were purchased from El-Nasr Chemicals Co. (Cairo, Egypt). Pharmaceutical dosage form, Cefotax^®^ vials (EIPICo., Tenth of Ramadan city, Egypt), containing cefotaxime sodium equivalent to 1000.0 mg CFX per vial were purchased from a local Egyptian market (produced by EIPICo., Egypt). Water was prepared in-house by double distillation. 

### 2.2. Instrumentation

All spectrophotometric measurements were carried out using a Schimadzu double-beam spectrophotometer, model UV-1201 (Schimadzu, Koyoto, Japan), equipped with 1 cm quartz cells and connected to a PC computer, programmed with UV probe software version 2.43. All chemometric models were implemented in MATLAB 8.2.0.701 (R2013b) software from (MathWorks, Natick, MA, USA). PLS-toolbox software version 2.1 was utilized for PLS and GA-PLS. 

### 2.3. Preparation of Standard Solutions

A stock standard solution of CFX was prepared by dissolving the pure drug in double-distilled water (final concentration, CFX 2.0 mg mL^−1^). A working standard solution of CFX (200 µg mL^−1^) was prepared from the stock solution by dilution with double-distilled water. Calibration standards were then prepared from the working solution in the same manner. All stock, working, and prepared calibration standards were refrigerated at 2–8 °C.

### 2.4. Acidic and Alkaline Degradation

For acidic degradation, 25 mL of 0.5 N HCl were added to 25 mL of stock standard solution (2 mg mL^−1^). For alkaline degradation, 25 mL of 0.5 N NaOH was added to 25 mL stock standard solution (2 mg mL^−1^). Both mixtures were heated in a boiling water bath under reflux for 1 h. The solutions were then left to cool and then neutralized to pH 7 using either 25 mL of 0.5 N NaOH or HCl, respectively. The two final solutions were combined to make 100 mL with double-distilled water and labeled to contain acidic and alkaline degradation products derived from 500 µg mL^−1^ as CFX. 

Complete degradation was confirmed by HPTLC experiments [2], through the disappearance of a spot corresponding to intact CFX and the appearance of one new spot corresponding to the degradation products using a mobile phase mixture composed of ethyl acetate:acetone:water:acetic acid (10:5:3:2, *v*/*v*, respectively).

### 2.5. Procedure 

#### 2.5.1. Construction of the Chemometric Models

A multilevel, multifactor design was utilized to construct the calibration and validation sets [35]. Seventeen samples were selected for the calibration set and eight samples for the validation set. A CFX working standard solution (200 µg mL^−1^) and each degradant solution were used to prepare a series of mixed dilutions with double distilled water composed of CFX/degradant mix separately in two sets, one set containing CFX/alkaline degradants and the other composed of a CFX/acidic degradant. The sample mixtures’ definite concentrations were selected according to analytesʹ linearity ranges, as illustrated in Table 1. Then, UV-probe software was used to scan the absorption spectra of the sample mixtures from 220 nm to 320 nm against a blank of distilled water. The intervals were selected to be 1 nm. The concentrations and absorbance values were inputted into MATLAB software in order to build the optimal CLS, PCR, PLS, and GA-PLS models.

#### 2.5.2. Pharmaceutical Dosage Form Analysis 

The contents of 3 different Cefotax^®^ vials were transferred into separate 500 mL volumetric flasks and dissolved in distilled water up to the mark to obtain a stock solution (2 mg mL^−1^). Working solutions (200 µg mL^−1^) were prepared by dilution from the previous stock solution using distilled water. Each Cefotax^®^ working solution (200 µg mL^−1^) was diluted into 10 mL volumetric flasks to a concentration of 10 µg mL^−1^ with distilled water (within the analytical working range). UV-probe software was used to scan the absorption spectra of the prepared solutions from 220 nm to 320 nm against a blank of distilled water at an interval of 1 nm.

## 3. Results

**Figure 2:**
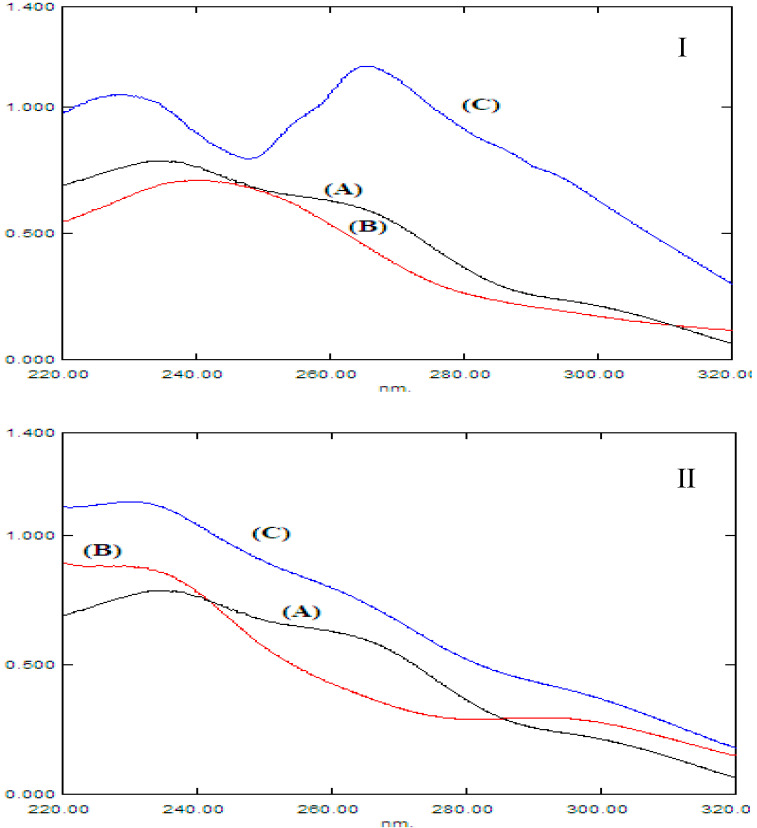
Absorption spectra of CFX together with its (**I**) acidic and (**II**) alkaline degradants for (**A**) 20 µg mL^−1^ CFX, (**B**) 20 µg mL^−1^ acidic degradants, and (**C**) both drug and degradants in the same mixture.

The UV-VIS scan revealed severe overlapping between the spectra of CFX and its acidic and alkaline degradants (Figure 2). Therefore, the direct imitation spectrophotometric methods did not succeed in resolving this overlap. Hence, four chemometric methods, namely, CLS, PCR, PLS, and GA-PLS, were developed for the simultaneous determination of CFX in the presence of its acidic degradants and its alkaline degradants, separately.

### 3.1. Spectral Zone Selection and Construction of a Calibration Matrix

For (CFX/acidic degradants) and (CFX/alkaline degradants), twenty-five sample mixtures were prepared, where seventeen samples were used for calibration and the remaining eight samples were used for validation. The spectral zone for both mixtures was selected in the range from 220 to 320 nm, with 1 nm intervals to obtain 101 spectral points. This spectral range was selected since the spectral points after 320 nm had low absorbance values close to zero, while those before 220 nm were noisy. 

The absorbance calibration set matrix for each mixture was (17 × 101), and its corresponding concentration matrix was (17 × 2) (Table 1). The data was introduced into the MATLAB software, which was then used to construct the developed chemometric models.

**Figure 3:**
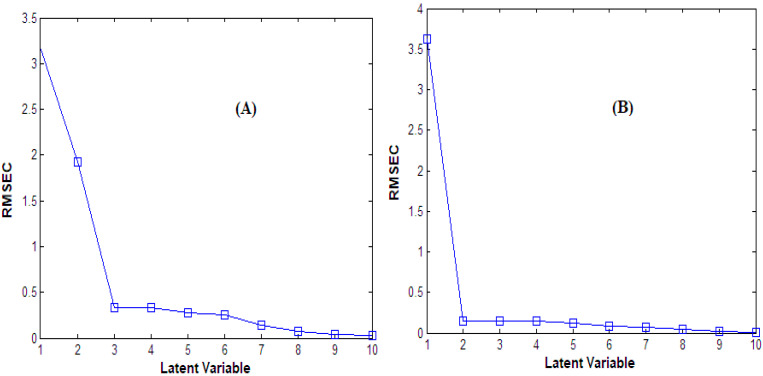
RMSEC plot of the cross-validation results of the calibration set as a function of the principal components number used to construct the PLS model for (**A**) the CFX/acidic degradants mixture and (**B**) the CFX/alkaline degradants mixture.

A pre-step for latent variable optimal number selection was performed for the PCR, PLS, and GA-PLS models. This selection was performed, leaving out one sample at a time from the seventeen spectra in the calibration set [36]. Three latent factors were found to be the optimum number for all methods in (CFX/acidic degradants), while two factors were the optimum number for all methods in (CFX/alkaline degradants) due to having the smallest prediction error value (Figure 3). 

**Figure 4:**
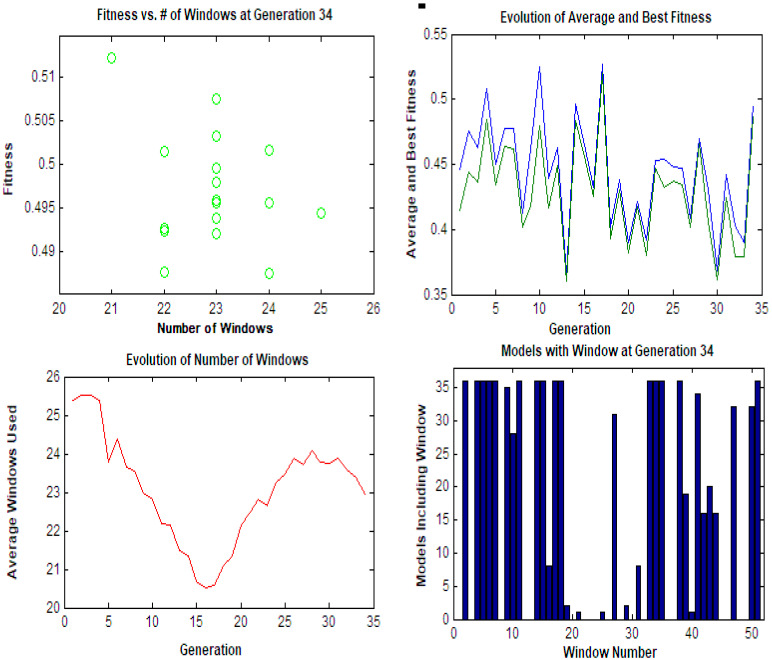
The parameters involved in the application of GA on the PLS model for the CFX/acidic degradant mixture.

For GA-PLS construction, the genetic algorithms technique was used to make the wavelength selection in the PLS regression model in order to minimize the number of spectral points used [34]. GA-PLS minimized the spectral points to 46.53% for (CFX/acidic degradants) for the PLS model and to 44.55% for (CFX/alkaline degradants) for the PLS model. All selected GA parameters are indicated in Table 2 and Figure 4. 

### 3.2. Validation of Chemometric Models

**Figure 5:**
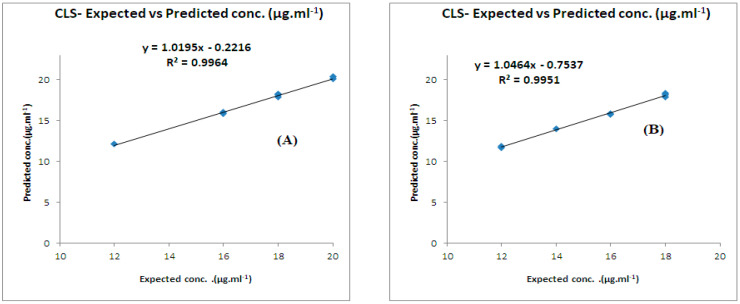
Actual versus predicted concentration plots for CLS models of (**A**) the CFX/acidic degradants mixture and (**B**) the CFX/alkaline degradants mixture.

**Figure 6:**
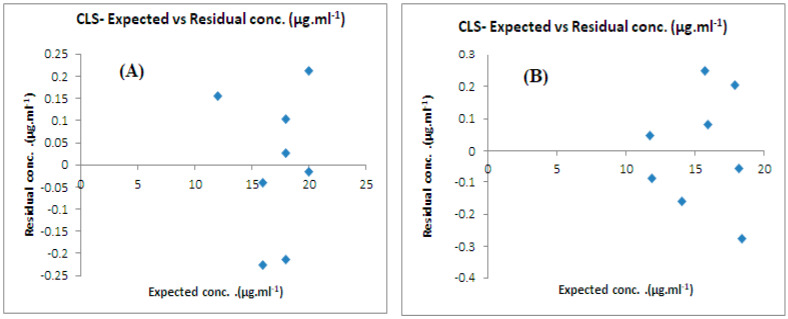
Actual versus residual concentration plots for CLS models of (**A**) the CFX/acidic degradants mixture and (**B**) the CFX/alkaline degradants mixture.

The predictive capabilities of the chemometric models were evaluated by several methods. RMSEP values were found to be (0.19, 0.29, 0.47, and 0.20) for the (CFX/acidic degradants) mixture and (0.21, 0.21, 0.21, and 0.22) for the (CFX/alkaline degradants) mixture for the CLS, PCR, PLS, and GA-PLS models, respectively, indicating the excellent accuracy and precision of the developed models (see Table 3 and Table 4). The prediction of CFX concentrations in samples, the calculation of their percentage recoveries, the standard deviation, and the RMSECV were also other tools to check the validity of the models (see Table 3 and Table 4). The curve plots that related the actual concentrations and the predicted concentrations in the validation samples were constructed, indicating good values of correlation coefficients (r) (Figure 5). Residual plots were also constructed between the concentration residuals and the expected ones, indicating a random allocation of residuals around zero (Figure 6). 

The results of all models were satisfactory. With the results compared, the CLS model for the CFX/acidic degradants mixture and the PCR model for the CFX/alkaline degradants mixture showed the lowest RMSEP and standard deviation values as well as the highest correlation coefficients (r). Hence, they were considered to be the best-developed models.

### 3.3. Pharmaceutical Formulation Application and Statistical Analysis

The developed methods were applied in the Cefotax^®^ vial assay. The results were satisfactory within acceptable limits and were statistically compared to the results obtained by another previously reported method [37]. The reported method is a spectrophotometric method based on the determination of CFX that reduced a silver-gelatin complex and yielded a yellow silver solution measured at the maximum wavelength of 352 nm [37]. No significant differences were found by applying the Student’s *t*-test and F-test at a 95% confidence level (Table 5).

### 3.4. Greenness Assessment of Analytical Methods

The core idea of green analytical chemistry (GAC) is to minimize the use of energy and hazardous chemical reagents for enhancing sustainability and lowering the ecological impacts of such methods [38]. Four main parameters within any analytical methodology affect their ecological impact. First of all, the procedure needed for sampling the materials to be tested. This step includes their online/offline sampling, the need for transportation, and sample preservation. Another main ecologically important parameter is the sample preparation steps involved in any new analytical methodology. The scale at which the analytes can be measured is where micro- and nano-analyses are better than macro-analyses, regardless of the sample treatment and organic solvents involved [39]. The type of reagents and the instrumentation used are considered two of the most important parameters. The amount, including the health and safety hazards of any reagent used within the analytical method, is considered. Water is the safest green solvent [40], and if possible, avoiding the use of chemicals is safer. The instrumentation has also received high appreciation in regard to energy utilization, generation of waste, and occupational hazards [41,42]. In general, spectroscopic methods, except MS, utilize less energy than HPLC and UHPLC. However, the waste generated from the UHPLC technique is much lower than HPLC. Therefore, when considering the proposed method according to the previous main parameters, they can be of added value for routine quality control of CFX in its marketed pharmaceutical formulations, especially in the light of the knowledge that their production has been currently growing with total sales’ expenditure of billions of USD [43]. This can give an indication about the number of analyses required in their QC procedures and their ecological impact.

**Figure 7:**
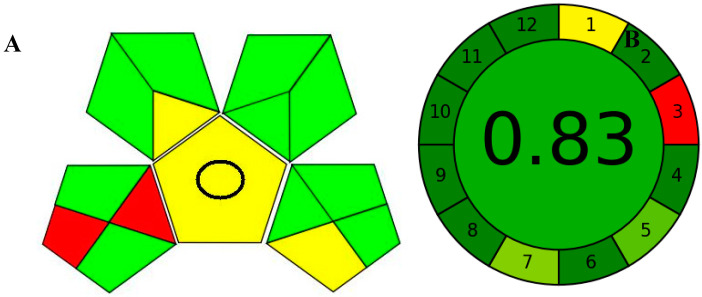
Evaluation of the greenness of the proposed methods using the GAPI (**A**) and the AGREE (**B**) assessment metrics.

The recent advances in green analytical chemistry (GAC) concepts have resulted in the development of a number of tools that are being used for the assessment of the greenness of newly developed analytical methodologies. Among those tools, the green analytical procedure index (GAPI) [44] and AGREE [45] have been the most widely applicable metrics recently. Both metrics use red-yellow-green color codes for evaluating the ecological impacts of different methodologies. Their methodologies are well established now and applied widely [46,47,48,49]. The proposed methods were assessed on the GAPI and AGREE metrics (Figure 7). As shown in Figure 7, the GAPI pictogram shows only two red zones, which correspond to off-line sampling and transportation, which correspond to zone 3 in the perimeter of the AGREE pictogram. These are obligatory requirements in pharmaceutical guidelines that separate the production from the quality control premises in the pharmaceutical industry. No organic solvents are being used by the proposed technique, only water. The overall score shown in the AGREE pictogram indicates the highest ecological compatibility and lowest impacts of the proposed methods.

### 3.5. Evaluation of the Proposed Methods against the Reported Ones

One of the useful tools when establishing new methodologies is to compare them to a previously reported one. As shown in Table 6, several techniques can be found within the literature survey for estimating CFX in the presence of its degradants, including HPLC, TLC, derivative spectroscopy, and densitometry. The proposed methods outperform the chromatographic techniques (HPLC and TLC methods) in terms of being more ecologically green, through avoiding the use of hazardous organic solvents and lowering energy consumption in HPLC instrumentation. Another added value is the fast analysis time, requiring just a few seconds, which hence provides higher throughput, which is a huge advantage in routine quality control of pharmaceuticals (Table 6). Moreover, the proposed methods can be applied for simultaneous determination of CFX in the presence of either acidic or alkaline degradation products, whereas the previously reported spectrophotometric methods were applied for CFX determination in the presence of only one degradation pattern (Table 6). Table 6 also shows the greenness assessment of the reported methods compared to the proposed methodologies on the GAPI scale. As shown, the proposed methods have the lowest ecological impact among all, as indicated by a fewer number of red zones and a greater number of green zones in their pictogram. Only method [21] showed a comparable GAPI pictogram; however, the method was used only with acidic degradants and needed sulfuric acid as a solvent, whereas the proposed methods only depend on water as the greenest ecological solvent and are capable of estimating the drug under study in the presence of both acidic and alkaline degradants.

## 4. Conclusions

In the present work, four new different multivariate chemometric analytical methods, namely, CLS, PCR, PLS, and GA-PLS, were developed and validated for the simultaneous determination of cefotaxime in the presence of its acidic or alkaline degradation products. The chemometric methods studied showed high sensitivity and good resolving power for separating the pure drug from its degradation products without the need for any separation steps. The proposed methods provide a more economical alternative to the other chromatographic methods for routine analysis of cefotaxime in both a pure form and a pharmaceutical dosage form without any interference from degradation products. The results of the validation parameters insured that all applied methods were linear, accurate, and precise, with no significant differences between the proposed and reported methods. Comparing the results of the CLS model for a CFX/acidic degradants mixture and the PCR model for a CFX/alkaline degradants mixture, they showed the lowest RMSEP (0.19 and 0.21) and standard deviation values (0.94 and 1.40), respectively. Hence, they were considered to be the best-developed models. Moreover, the developed methods excel at being simple, easy, green, and fast, unlike chromatographic methods, which are more complex, expensive, time-consuming, and not environmentally friendly in most cases. The prediction capabilities of the developed models were compared and proved to be high.

## Figures and Tables

**Table 1:** Concentrations of laboratory-prepared mixtures of CFX with its acidic and alkaline degradants used in the calibration and validation sets.

CFX/Acidic Degradants Mixture			CFX/Alkaline Degradants Mixture		
Sample No.	CFX (μg mL^−1^)	Acidic Degradant (μg mL^−1^)	Sample No.	CFX (μg mL^−1^)	Alkaline Degradant (μg mL^−1^)
1	16	25	1 *	16	15
2 *	16	15	2	16	5
3	12	15	3	12	5
4 *	12	35	4	12	25
5	20	20	5	20	10
6	14	35	6	14	25
7	20	25	7	20	15
8	16	20	8	16	10
9	14	20	9	14	10
10	14	30	10	14	20
11 *	18	35	11	18	25
12	20	30	12	20	20
13 *	18	25	13 *	18	15
14 *	16	35	14	16	25
15 *	20	35	15	20	25
16 *	20	15	16	20	5
17	12	30	17	12	20
18	18	15	18 *	18	5
19 *	12	25	19 *	12	15
20	16	30	20 *	16	20
21	18	30	21 *	18	20
22	18	20	22	18	10
23	14	15	23 *	14	5
24	12	20	24 *	12	10
25	14	25	25	14	15

* Concentrations of mixtures used in the validation set.

**Table 2:** Parameters of the genetic algorithm.

Parameter	(CFX/Acidic Degradants) GA-PLS Model	(CFX/Alkaline Degradants) GA-PLS Model
Population size	36	36
Maximum generations	34	34
Mutation rate	0.005	0.005
The number of variables in a window (window width)	2	2
Percent of population the same at convergence	80	80
Percent wavelengths used at initiation	50	50
Crossover type	Double	Double
Maximum number of latent variables	3	2
Cross validation	Random	Random
Number of subsets to divide data into for cross validation	4	4

**Table 3:** The prediction recoveries of the validation set samples by the four proposed multivariate methods for (CFX/alkaline degradants).

Mix. No.	CFX Actual Conc. (μg mL^−1^)	CFX/Acidic Degradants Mixture				Mix. No.	CFX Actual Conc. (μg mL^−1^)	CFX/Alkaline Degradants Mixture			
		CLS	PCR	PLS	GA-PLS			CLS	PCR	PLS	GA-PLS
2	16	99.15	98.46	98.95	99.03	1	16	98.31	98.31	98.31	98.16
4	12	101.39	101.60	101.62	102.23	13	18	102.14	102.12	102.12	101.77
11	18	101.29	99.52	96.44	98.97	18	18	100.87	100.80	100.80	101.16
13	18	100.86	98.47	97.86	99.21	19	12	97.78	97.80	97.80	97.99
14	16	100.30	99.66	97.37	101.94	20	16	99.40	99.44	99.43	99.07
15	20	101.90	98.95	95.95	99.32	21	18	99.33	99.37	99.36	98.59
16	20	100.76	98.10	98.58	98.79	23	14	100.41	100.37	100.37	100.99
18	18	99.53	96.96	97.53	99.57	24	12	99.00	98.98	98.98	99.52
Mean		100.65	98.97	98.04	99.88			99.65	99.65	99.65	99.66
SD		0.94	1.36	1.76	1.38			1.42	1.40	1.40	1.46
RSD		0.93	1.38	1.79	1.38			1.43	1.40	1.41	1.45
RMSEP		0.19	0.29	0.47	0.20			0.21	0.21	0.21	0.22

**Table 4:** Statistical parameters for the four developed multivariate models for the CFX/alkaline degradant mixture.

Parameter	CLS	PCR	PLS	GA-PLS
Wavelength range (nm) *	220–320			
Linear range (μg.ml^–1^)	12–20			
RMSECV	0.146	0.146	0.146	0.162
LV number	-	-	2	2
Mean (%)	99.65	99.64	99.64	99.62
RSD (%)	1.42	1.39	1.41	1.47
R **	0.9951	0.9953	0.9952	0.9929
Slope **	1.046	1.046	1.046	1.028
Intercept **	−0.754	0.751	0.750	−0.479

* Trial-and-error method based on RMSECV selection. ** Calculated for the actual and predicted values of the validation set.

**Table 5:** Statistical comparison between the results obtained by the developed methods and the reported method [37] for the assay of CFX in Cefotax^®^ vials.

		CFX/Acidic Degradation Method				CFX/Alkaline Degradation Method			
Parameter	Reported Method [37]	CLS	PCR	PLS	GA-PLS	CLS	PCR	PLS	GA-PLS
Mean	99.92	99.77	99.83	100.11	100.21	99.74	99.58	99.88	99.84
V	0.13	0.15	0.16	0.07	0.14	0.24	0.25	0.34	0.26
N	8	5	5	5	5	5	5	5	5
Student’s *t*-test (2.201) ^a^	--	0.71	0.42	1.01	1.39	0.77	1.43	0.15	0.33
F-test (7.85) ^a^	--	1.15	1.23	1.86	2.64	1.85	1.92	2.62	2.00

^a^ Figures in parentheses are the corresponding tabulated values at *p* = 0.05.

**Table 6:** Comparison between the proposed and reported methods applied for the simultaneous determination of CFX and its degradation products.

	Technique Applied	Analysis Time	Solvents Used	Linearity Range	Degradation Product	Application	GAPI Assessment
Proposed method	Multivariate spectrophotometry	Seconds	Water	12–20 µg mL^−1^	Acidic or alkaline	Pharmaceutical dosage forms	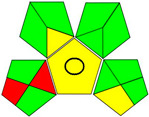
Reported [21]	Second derivative spectroscopy	Seconds	Sulfuric acid	4–24 µg mL^−1^	Acidic only	Pharmaceutical dosage forms	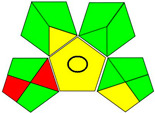
Reported method [20]	Derivative spectroscopy	Seconds	Water	5–40 µg mL^−1^	Acidic only	Pharmaceutical dosage forms	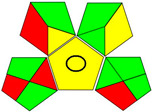
	Densitometric	About 10 min	Methanol:acetic acid	2–12 µg/spot	Acidic only	Pharmaceutical dosage forms	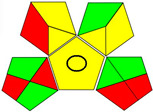
Reorted method [14]	FSQ spectrophotometric	Seconds	Acetonitrile:water (10:90)	10–22 µg mL^−1^	Alkaline only	Pharmaceutical dosage forms	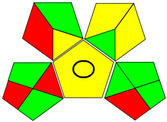
	HPLC	5 min	Ammonium acetate:acetonitrile	5–20 µg mL^−1^	Alkaline only	Pharmaceutical dosage forms	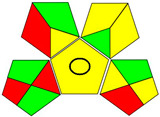
Reported method [18]	HPLC	15 min	Methanol:phosphate buffer	0.5–1.5 µg mL^−1^	Acidic, alkaline, or oxidative	Pharmaceutical dosage forms	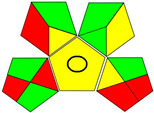
Reported method [17]	HPLC	10 min	Methanol:acetonitrile:buffer	51–360 µg mL^−1^	Acidic, alkaline, or oxidative	Pharmaceutical dosage forms	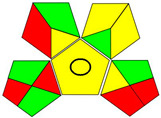
Reported method [15]	TLC	More than 10 min	Benzene and Methanol:acetic acid	100–600 ng/spot	Acidic, alkaline, or oxidative	Pharmaceutical dosage forms	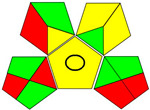

## Data Availability

All data are available upon request from the corresponding author.

## Sample Availability

Samples of the compounds are not available from the authors.
